# Evasion of Antiviral Innate Immunity by Theiler's Virus L* Protein through Direct Inhibition of RNase L

**DOI:** 10.1371/journal.ppat.1003474

**Published:** 2013-06-27

**Authors:** Frédéric Sorgeloos, Babal Kant Jha, Robert H. Silverman, Thomas Michiels

**Affiliations:** 1 Université Catholique de Louvain, de Duve Institute, Brussels, Belgium; 2 Department of Cancer Biology, Lerner Research Institute, Cleveland Clinic, Cleveland, Ohio United States of America; McMaster University, Canada

## Abstract

Theiler's virus is a neurotropic picornavirus responsible for chronic infections of the central nervous system. The establishment of a persistent infection and the subsequent demyelinating disease triggered by the virus depend on the expression of L*, a viral accessory protein encoded by an alternative open reading frame of the virus. We discovered that L* potently inhibits the interferon-inducible OAS/RNase L pathway. The antagonism of RNase L by L* was particularly prominent in macrophages where baseline oligoadenylate synthetase (OAS) and RNase L expression levels are elevated, but was detectable in fibroblasts after IFN pretreatment. L* mutations significantly affected Theiler's virus replication in primary macrophages derived from wild-type but not from RNase L-deficient mice. L* counteracted the OAS/RNase L pathway through direct interaction with the ankyrin domain of RNase L, resulting in the inhibition of this enzyme. Interestingly, RNase L inhibition was species-specific as Theiler's virus L* protein blocked murine RNase L but not human RNase L or RNase L of other mammals or birds. Direct RNase L inhibition by L* and species specificity were confirmed in an *in vitro* assay performed with purified proteins. These results demonstrate a novel viral mechanism to elude the antiviral OAS/RNase L pathway. By targeting the effector enzyme of this antiviral pathway, L* potently inhibits RNase L, underscoring the importance of this enzyme in innate immunity against Theiler's virus.

## Author Summary

Theiler's virus is a murine picornavirus (same family as poliovirus) which has a striking ability to establish persistent infections of the central nervous system. To do so, the virus has to counteract the immune response of the host and particularly the potent response mediated by interferon. We observed that a protein encoded by Theiler's virus, the L* protein, inhibited the RNase L pathway, one of the best-characterized pathways mediating the antiviral IFN response. In contrast to previously identified viral antagonists of this pathway, L* was found to act directly on RNase L, the effector enzyme of the pathway. L* activity was found to be species-specific as it inhibited murine but not human RNase L. We confirmed the species-specificity and the direct interaction between L* and RNase L *in vitro*, using purified proteins. Acting at the effector step in the pathway allows L* to block RNase L activity efficiently. This suggests that RNase L is particularly important to control Theiler's virus replication *in vivo*. Another virus, mouse hepatitis virus (MHV), was recently shown to interfere with RNase L activation. Theiler's virus and MHV share a marked tropism for macrophages which may suggest that the RNase L pathway is particularly important in this cell type.

## Introduction

Control of infectious agents is a major challenge in the homeostasis maintenance of organisms. Among vertebrates, interferons (IFNs) are the most important cytokines aimed to restrict viral replication and spread. IFNs induce the expression of numerous proteins that are responsible for their antiviral activities. The 2′–5′ oligoadenylate synthetase (OAS)/RNase L pathway is one of the best-characterized IFN effector pathways [1]. This tightly regulated antiviral pathway operates by controlling RNA integrity. Triggering of the OAS/RNase L pathway depends on the production of dsRNA, a by-product of viral replication [2]. Viral dsRNA stimulates OAS enzymes to catalyze the conversion of ATP into 2′–5′ oligoadenylates (2–5A) [3]. Newly produced 2–5A function as direct activators of the latent inactive RNase L [4]. 2–5A binding on the N-terminal ankyrin domain of the monomeric enzyme induces its oligomerization and activation [5], [6]. Enzymatically active RNase L subsequently cleaves single-stranded regions of viral and cellular RNA, leading to the inhibition of protein synthesis, decreased viral replication and ultimately cell apoptosis. Moreover, RNase L was reported to be involved in amplification of IFN signaling [7]. Antiviral activity of the OAS/RNase L pathway was demonstrated for a wide variety of viruses including Encephalomyocarditis virus (EMCV) [8], Herpes Simplex virus-1 (HSV-1) [9], [10], Vaccinia virus (VV) [11], Coxsackie B4 virus [12], West Nile virus [13] and Coronaviruses [14], [15].

DNA and RNA viruses evolved different strategies to counteract RNase L activity. For example, IFN-resistant genotypes of Hepatitis C virus (HCV), accumulated fewer RNase L cleavage sites as compared to IFN-sensitive HCV genotypes [16], [17]. Poliovirus delays RNase L activation by bearing a cleavage-resistant RNA sequence that interferes with endoribonuclease activity [18], [19]. EMCV was shown to upregulate the expression of RLI, a cellular protein that antagonizes 2–5A binding and RNase L activation [20]. The product of gene 7 of the transmissible gastroenteritis virus (TGEV), a swine coronavirus, was shown to be responsible for the inhibition of cellular RNA degradation. It is suggested that a complex comprising the gene 7 product and the cellular phosphatase PP1c is responsible for RNase L inhibition through 2–5A dephosphorylation [14]. More recently, mouse hepatitis virus (MHV) was shown to encode a phosphodiesterase which cleaves 2–5A [15]. RNase L antagonism was also reported for DNA viruses including HSV, SV40 and VV, via the production of inactive 2–5A analogs during infection. However, the identity of these 2–5A related compounds and the mechanism of their production are unknown [21]–[24]. Interestingly, RNase L germline mutations have been associated with increased risks of prostate cancer as well as head and neck, uterine cervix and breast cancer [25], [26].

Theiler's murine encephalomyelitis virus (TMEV or Theiler's virus), a member of genus *Cardiovirus* within the *Picornaviridae* family, is a natural pathogen of mice displaying a rare incidence of spontaneous neuro-invasion [27]. Persistent strains of Theiler's virus are responsible for a biphasic CNS infection. After initial replication of the virus in gray matter of the brain, the virus migrates to the spinal cord white matter where it mainly infects macrophages and oligodendrocytes [28], [29]. In the spinal cord, the virus persists lifelong in spite of a strong innate and adaptive immune response and triggers a chronic demyelinating disease reminiscent of human multiple sclerosis [30], [31]. Viral persistence and subsequent demyelination require the expression of L*, a 156 aminoacid-long viral accessory protein encoded by an alternative open reading frame (ORF) that overlaps the main viral ORF [32]–[34]. Mechanisms used by L* to promote viral persistence remain poorly understood. L* was shown to drive sustained TMEV replication in macrophage cell lines [35], [36]. This effect was shown to be specific to macrophages and was not observed in other cell types, such as neurons or fibroblasts [37]. Facilitation of TMEV replication in macrophages is probably significant for TMEV persistence *in vivo*, as these cells bear the major viral load during chronic infection [28]. Increased viral replication in macrophages was reported to result from anti-apoptotic activity [32], [38].

We recently observed that, in infected cells, L* was partitioned between the cytoplasm and the mitochondrial outer membrane [39]. Since mitochondrial localization fitted well with the reported anti-apoptotic role of L*, we tested whether L* anti-apoptotic activity was responsible for the observed facilitation of viral replication in macrophages. We observed very little if any activity of L* on apoptosis, but a prominent inhibition of the OAS/RNase L pathway. Our work elucidated an important function of the L* protein. We show that L* antagonizes RNase L activity through a direct protein-protein interaction. This is the first example of viral protein acting at the effector step of this pathway. Moreover, our data show that L* acts in a species-specific fashion.

## Results

### L* protein prevents rRNA degradation in infected cells

**Figure 1:**
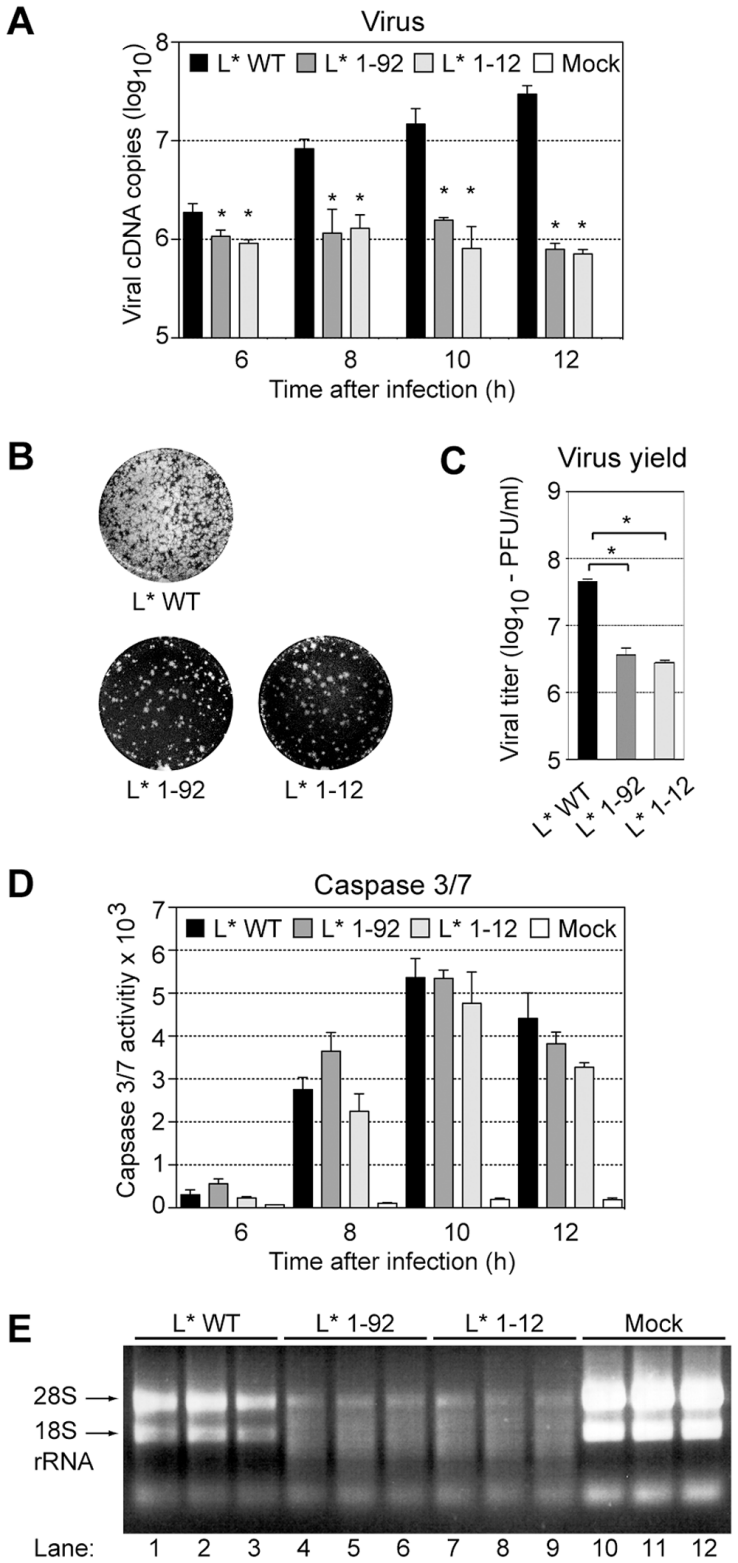
L* fails to block apoptosis but prevents RNA degradation in infected macrophages. A–C. Impaired replication of L*-mutant viruses in J774-1 macrophages: J774-1 cells were infected with VV18 (L* WT)(black columns) or L*-mutant viruses TM770 (L* 1–92)(dark grey columns) and FS58 (L* 1–12)(light grey columns) at a MOI of 20, or left uninfected (white columns). Viral genomes were quantified at indicated times, by quantitative RT-PCR. Values for mock samples were lower than the detection limit (10 cDNA copies) (A). Infectious virus yield in the supernatant was measured by plaque assay. Panel B shows plaques formed by equivalent supernatant dilutions and panel C shows the quantification of the plaques (mean and SD). D. Caspase 3/7 activities in J774-1 infected macrophages were quantified at indicated times on the basis of their ability to cleave a luminogenic substrate. Values represent the mean of relative light unit (RLU) +/− SD calculated from a representative experiment performed in triplicate. E. Cellular RNA degradation: Total cell RNA extracted from J774-1 macrophages that were either mock-infected (lanes 10–12) or infected for 10 h with VV18 (L* WT, lanes 1–3), TM770 (L* 1–92, lanes 4–6) or FS58 (L* 1–12, lanes 7–9) was run on a native 1% agarose gel and stained with ethidium bromide (bands corresponding to 28S and 18S rRNA are indicated).

In an attempt to test the influence of L* on apoptosis in macrophages, J774-1 cells were infected with the wild-type VV18 virus (L* WT) or with the L*-mutant viruses, TM770 and FS58, which bear a stop codon in the L* ORF at codons 93 (L* 1–92) and 13 (L* 1–12), respectively. These mutations were previously shown to affect L* protein production and the establishment of persistent CNS infections by Theiler's virus [34]. As expected, replication of the wild-type virus, measured by quantitative RT-PCR, exceeded that of the mutant viruses. The difference between wild-type and mutant virus replication was about 2-fold at 6 hours post-infection and rose over time to reach about 40-fold at 12 hours post-infection (Fig. 1A). Accordingly, infectious virus yield, quantified by plaque assay, was one log higher in the case of the wild-type virus (Fig. 1B–C). Yet, TUNEL staining of infected cells and caspase 3/7 assays failed to show any reproducible influence of L* on J774-1 cell apoptosis (Fig. 1D and S1). These observations suggested that apoptosis was not directly linked to the impaired replication of L*-mutant viruses in macrophage cell lines. Interestingly, we noticed that cellular RNA isolated from cells infected with the L*-mutant viruses exhibited extensive degradation, in contrast to RNA isolated from cells infected with the wild-type virus or from mock-infected cells (Fig. 1E). These data suggested that L* inhibits a cellular ribonuclease (RNase) activated following virus infection. We hypothesized that this RNase was RNase L, an effector of the IFN response [40].

### TMEV L* protein inhibits the OAS/RNase L pathway

**Figure 2:**
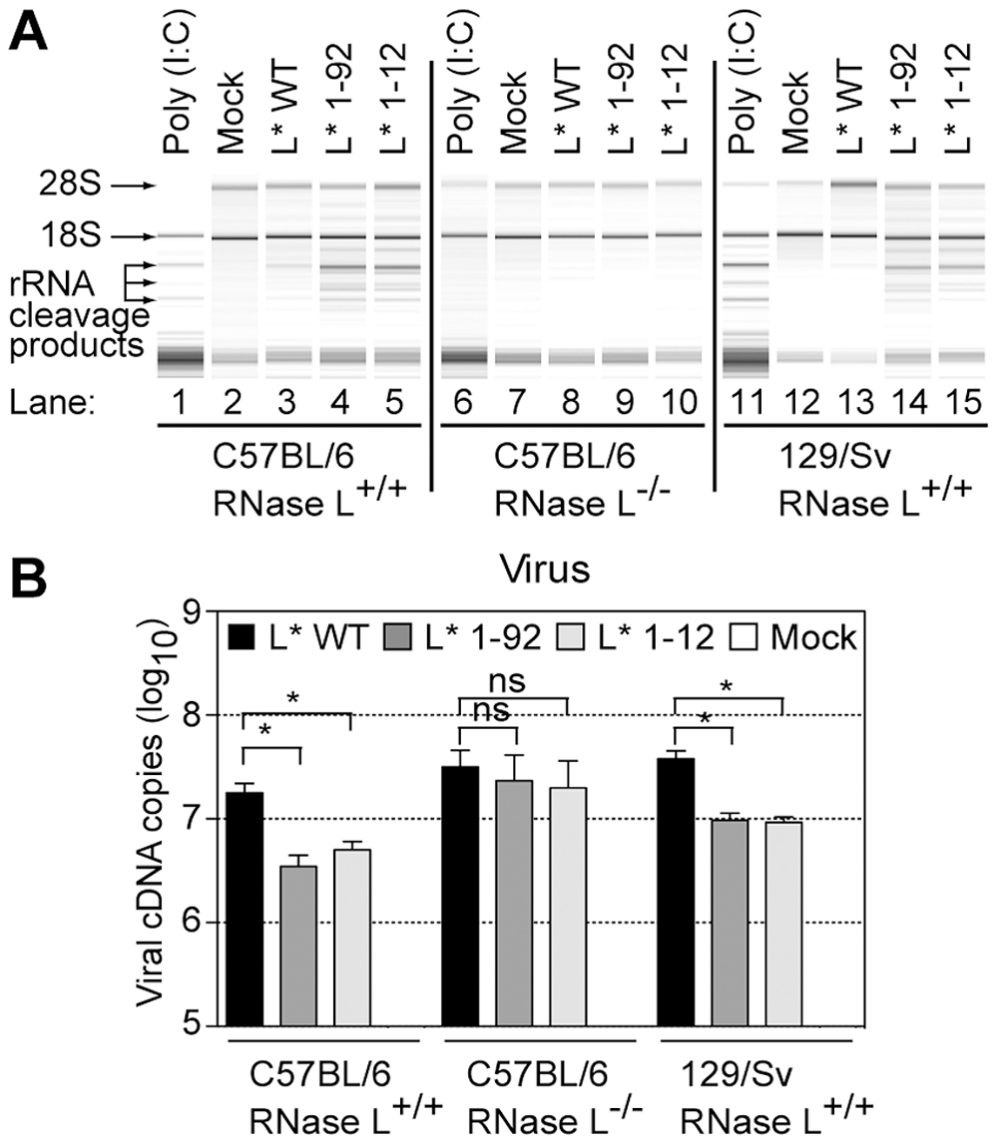
Theiler's virus L* protein inhibits the 2–5A/RNase L pathway in infected peritoneal macrophages. A. Peritoneal macrophages prepared from RNase L ^−/−^ (central panel) or RNase L^+/+^ (left) C57BL/6 mice and, as an additional control, from RNase L^+/+^ 129/Sv mice (right), were mock-infected or infected at a MOI of 20 with VV18 (L* WT), TM770 (L* 1–92) or FS58 (L* 1–12). Nine hours post infection, total cell RNA was extracted and analyzed on RNA chips. For control purposes, peritoneal macrophages were also transfected with 2.5 µg/ml poly(I:C) for 7 h before total cell RNA extraction. Prominent rRNA cleavage products are indicated. B. Replication levels of wild-type or L*-mutant viruses in peritoneal macrophages. Peritoneal macrophages isolated from indicated mouse strains were plated for four days and infected as in (A). Viral RNA was quantified by quantitative RT-PCR. Histograms show the mean and SD of viral cDNA copies detected in samples from a representative triplicate infection experiment. Values for mock samples were lower than the detection limit (10 cDNA copies). The experiment was repeated twice, using two independent productions of viruses and macrophages.

To test whether RNase L was the nuclease responsible for RNA degradation in cells infected with L*-mutant viruses, we compared rRNA degradation in peritoneal macrophages prepared from RNase L^−/−^ and RNase L^+/+^ mice and infected with 20 plaque-forming units (PFU) per cell of virus VV18 (L* WT), TM770 (L* 1–92) or FS58 (L* 1–12). As a control of RNase L activity, RNase L^+/+^ and RNase L^−/−^ macrophages were also transfected with polyinosinic∶polycytidylic acid (poly(I:C)). Nine hours post-infection or 7 hours after poly(I:C) transfection, RNA was extracted from cells and separated on RNA chips (Fig. 2A). As expected, poly(I:C) transfection triggered RNA degradation in RNase L^+/+^ (Fig. 2A, lanes 1, 11) but not in RNase L^−/−^ (Fig. 2A, lane 6) macrophages. After infection, RNA degradation was detectable in RNase L^+/+^ cells infected with the L*-mutant viruses (Fig. 2A, lanes 4, 5, 14, 15) but not in cells infected with the wild-type virus, in agreement with the observations made in J774-1 macrophages. In RNase L^−/−^ macrophages, infection with the L*-mutant viruses failed to trigger RNA degradation (Fig. 2A, lanes 9, 10). These results show that RNase L is the nuclease responsible for infection-induced RNA degradation and that TMEV L* inhibits this activity.

Viral replication in infected cells was monitored by quantitative RT-PCR. In RNase L^+/+^ peritoneal macrophages, viral RNA levels were significantly lower (3 to 5-fold) for the L*-mutant viruses than for the wild-type virus. In contrast, in RNase L^−/−^ peritoneal macrophages, L*-mutant viruses replicated at the same level as the wild-type virus (Fig. 2B). Indirect immunofluorescent labeling of the VP1 capsid protein confirmed the higher replication level of the wild-type virus in RNase L^+/+^ cells (Fig. S2). These data show that the reduced fitness of L*-mutant viruses in macrophages mostly results from the inability of these viruses to counteract RNase L activity.

### Inhibition of the OAS/RNase L pathway by L* is not restricted to macrophages

Basal expression of enzymes involved in the IFN-inducible 2–5A/RNase L pathway can vary according to the cell type. For example, constitutive RNase L expression was shown to be higher in RAW264.7 macrophages than in L929 fibroblasts [41]. Therefore, we hypothesized that the phenotype of L*-mutant viruses was more prominent in macrophages because these cells express higher basal levels of OAS and/or RNase L than fibroblasts. In this hypothesis, L* activity would also be effective in fibroblasts, after induction of the OAS/RNase L pathway by IFN treatment.

We first compared, by quantitative RT-PCR, the abundance of mRNA encoding OASL2 and RNase L in the L929 (fibroblasts) and J774-1 (macrophages) cell lines, and in primary macrophages. RNase L expression did not vary much (Fig. S3). In contrast, basal *Oasl2* expression was 100-fold lower in L929 than in J774-1 cells and about 20-fold lower than in peritoneal macrophages. However, *Oasl2* expression in L929 cells could be induced to levels similar to those found in J774-1 cells, after treatment of the cells with 5 units/ml of IFN-β (Fig. S3).

**Figure 3:**
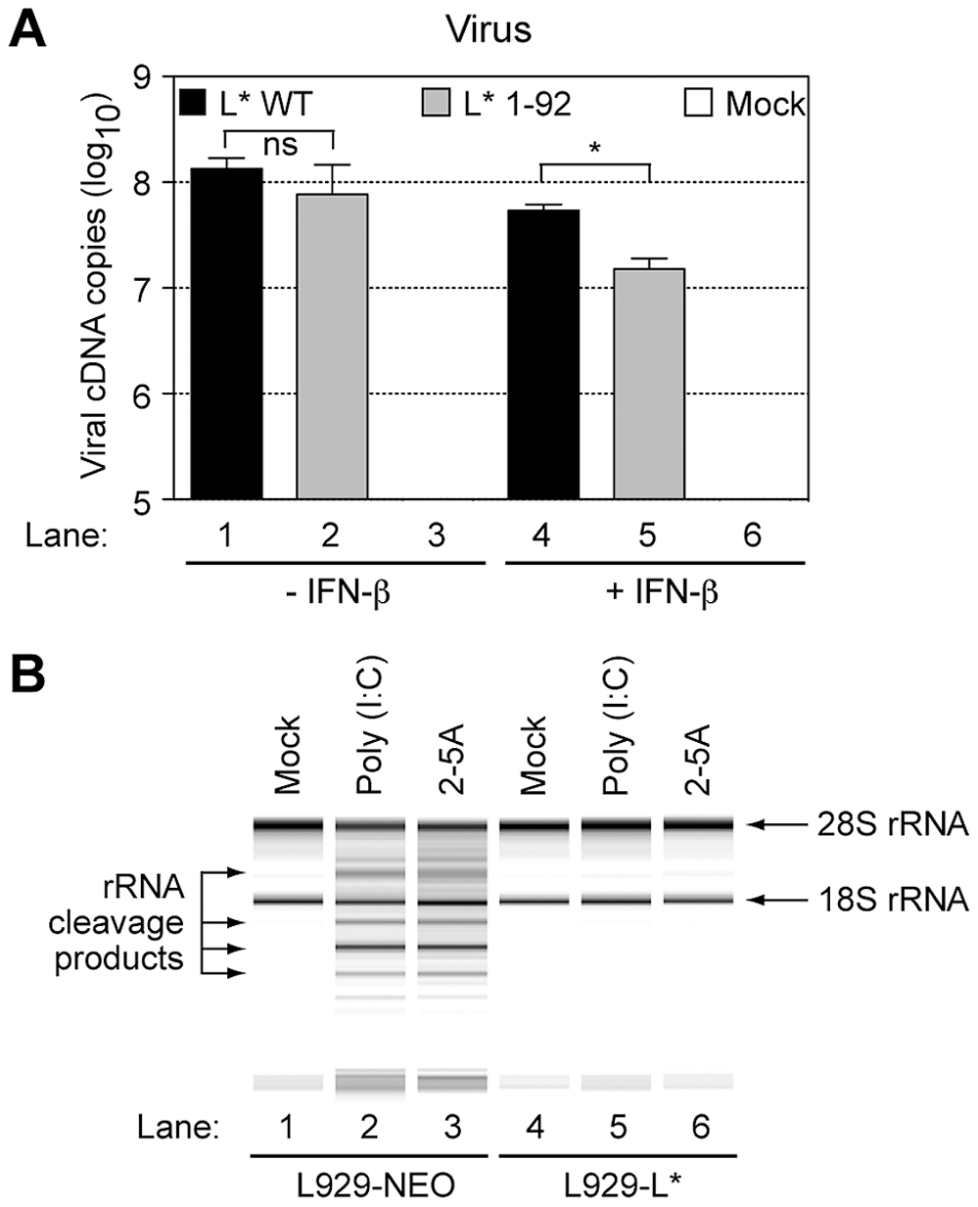
L* can act on non-macrophage cell lines and in absence of other viral components. A. L* inhibition of the 2–5A/RNase L pathway is not restricted to macrophages: L929 cells were incubated with 5 U/ml of murine IFN-β for 24 h or left untreated prior to infection with 2 PFU per cell of indicated viruses. Histograms show the mean +/− SD of viral genome copies detected by quantitative RT-PCR in RNA samples isolated 16 h after infection. Values for mock samples were lower than the detection limit (10 cDNA copies). B. L* inhibits the 2–5A/RNase L pathway in the absence of other viral components: L929 cells stably expressing L* (L929-L*) or the empty vector (L929-NEO) were primed with 5 U/ml of murine IFN-β and transfected with poly(I:C) for 7 hours. Alternatively, the cells were directly transfected without priming, with crude 2–5A at a concentration of 5 uM. Total cell RNA was then isolated and analyzed on RNA chips.

Then, we tested whether L* activity was detectable in IFN-β-treated L929 cells. Mock-treated or IFN-β-treated L929 cells were infected with 2 PFU per cell of viruses KJ6 (L* WT) and FS57 (L* 1–92). Total RNA was extracted 16 hours post-infection and viral replication was followed by quantitative RT-PCR. In the absence of IFN pre-treatment, no significant difference was observed between replication levels of the wild-type and the L*-mutant virus. In contrast, in IFN-β-treated cells, a small (3.5-fold) but significant reduction of viral RNA replication was observed for the mutant, compared to the wild-type virus (Fig. 3A). We concluded that L* protein activity can be detected in fibroblasts, after IFN-β treatment.

When RNA extracts from infected cells were run on RNA chips, RNA degradation was visible in samples from IFN-β-primed L929 cells infected with the L*-mutant virus but not in samples from cells infected with the wild-type virus (data not shown). These results indicate that the inhibition of the OAS/RNase L pathway by TMEV L* is effective in non-macrophage cell lines, after pretreatment of the cells with low doses of type-I IFN.

### L* ectopic expression prevents poly(I:C) and 2–5A-mediated rRNA degradation

We tested whether expression of L*, in the absence of other viral components, could inhibit RNase L activity. Therefore, L929 cells were transduced with retroviral vectors expressing L* (L929-L*) or transduced with the empty vector (L929-NEO). We first tested whether L*, expressed in trans, could rescue the replication of L*-mutant viruses in IFN-β-treated cells. As shown in Fig. S4A, replication of FS57 (L* 1–92) was lower than that of KJ6 (L* WT) in control L929-NEO cells but did not differ from that of the wild-type virus in L929 cells constitutively expressing L*. Accordingly, ectopic expression of L* in the J774-1 macrophage cell line strongly increased replication of an L* mutant virus and inhibited RNA degradation in infected cells (Fig. S4B). Thus, ectopically expressed L* both rescued the replication of L*-mutant viruses and inhibited RNA degradation in infected cells.

We next used the transduced L929 cells to examine the step at which L* inhibited the OAS/RNase L pathway. Activation of the IFN-inducible RNase L is a multistep mechanism involving: i) IFN-mediated OAS gene transcription upregulation, ii) dsRNA-triggered activation of OAS enzymatic activity, iii) synthesis of 2–5A by these enzymes, and finally, iv) homodimerization and activation of RNase L through 2–5A binding. Given the basic nature of L* (calculated pI = 10.7), we hypothesized that this protein might act by sequestering double-stranded RNA, thereby preventing the activation of OAS enzymatic activity.

Poly(I:C) was transfected in IFN-β-primed L929-L* and L929-NEO cells. Seven hours post-transfection, total cell RNA was extracted and analyzed on RNA chips (Fig. 3B). Poly(I:C)-triggered RNA degradation was observed in samples from control cells (Fig. 3B, lane 2), but not in samples from L*-expressing cells (Fig. 3B, lane 5).

Next, L929-NEO and L929-L* cells were transfected with 2–5A, to bypass the OAS step in the RNase L pathway. While a significant activation of RNase L occurred in control cells (Fig. 3B, lane 3), no RNA degradation was detected in L*-expressing cells (Fig. 3B, lane 6), indicating that L* acted downstream of oligoadenylate synthesis by OAS enzymes. Accordingly, measurements of 2–5A concentrations in L929-NEO and L929-L* cells transfected with poly(I:C) failed to reveal any influence of L* on 2–5A production (Fig. S4C).

### RNase L inhibition is mediated by the L* protein

**Figure 4:**
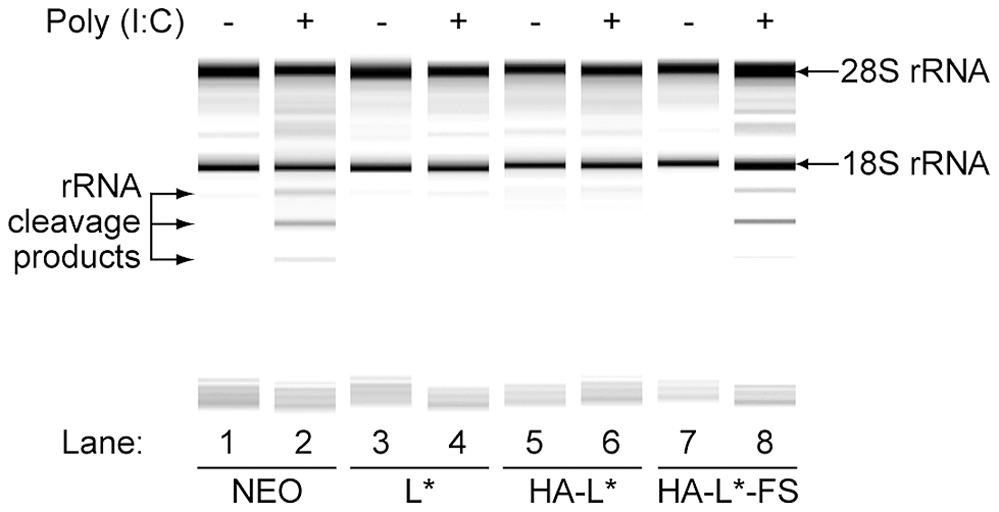
RNase L inhibition is due to L* protein. L929 expressing the empty vector (Neo), wild-type L* (L*), HA-tagged L* (HA-L*) and the frame-shifted HA-L* (HA-L*-FS) were primed with IFN-β and transfected with poly(I:C) or left untreated. Total cellular RNA was isolated 7 hours after transfection and separated on RNA chips. Intact 28S and 18S ribosomal RNA as well as characteristics rRNA cleavage products are indicated.

An atypical mechanism of RNase L inhibition has been discovered in the case of poliovirus, the prototypical member of the *Picornaviridae* family [18]. In this case, RNase L antagonism involved a cleavage-resistant RNA structure formed by the 3C-coding region of the poliovirus genome, that competitively inhibits RNase L activity [19]. We thus tested, in the case of TMEV, whether inhibition of the OAS/RNase L pathway observed in cells expressing L* was due to the expression of L*-coding RNA or to the L* protein itself. To this end, we constructed retroviral vectors encoding either a N-terminally HA-tagged L* or a frameshifted HA-L* obtained by deleting a single guanine nucleotide in the first codon of the construct. The presence of the N-terminal HA tag thus allowed to introduce the frameshift without modifying the RNA sequence in the L* moiety of the construct. Cells expressing equivalent levels of the two constructs were generated and incubated in presence of IFN-β to induce sufficient expression of the OAS enzymes. Then, RNA degradation was assessed 7 hours after poly(I:C) transfection. As shown in Fig. 4, RNA was protected from degradation in cells expressing HA-L* (lane 6), but not in control cells (lane 2) or in cells expressing the frameshifted HA-L* (lane 8). Thus, unlike for poliovirus, RNase L antagonism by TMEV involves a viral protein.

### TMEV L* protein interacts with the murine RNase L during infection

**Figure 5:**
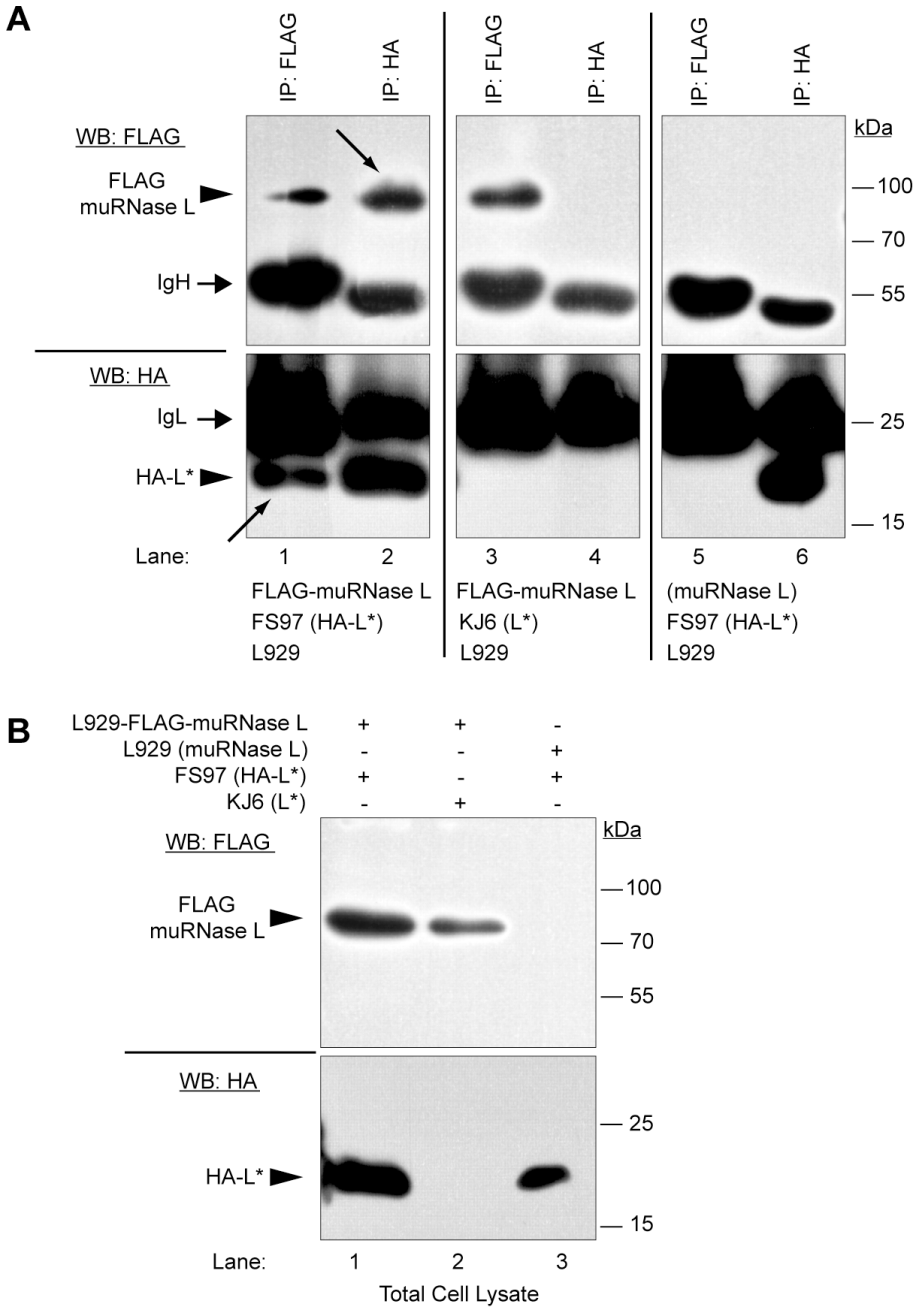
TMEV L* and muRNase L proteins interact in infected murine cells. L929 cells stably expressing FLAG-muRNase L were infected with FS97, a recombinant TMEV coding for HA-L*. Cell lysates were prepared and immunoprecipitated with indicated antibodies (IP:FLAG or IP:HA). A. Immunocomplexes were analyzed by Western blotting with the indicated antibodies (WB:HA or WB:FLAG). Arrows point to the coimmunoprecipitated proteins. B. As a control cell lysates corresponding to 5% of the input used for immunoprecipitations were analyzed by immunoblot using the same antibodies as in A. IgH, immunoglobulin heavy chain; IgL, immunoglobulin light chain.

The above data led us to hypothesize that RNase L could be inactivated through its association with L*. Because of the absence of reliable anti-murine RNase L antibodies and of tagged RNase L clones, we cloned the murine RNase L from SJL/J brain cDNA (Genbank accession JX443419) and verified that the cloned RNase L was catalytically active and 2–5A-dependent (data not shown). We next introduced a N-terminal FLAG tag in the construct and also tested its activity. We also constructed a TMEV recombinant virus, called FS96, expressing a N-terminally HA-tagged L* protein (HA-L*) and verified that the HA-tagged virus conserved the ability to persist in the CNS of susceptible mice and, hence, that the tag did not affect the function of L* *in vivo* (Fig. S5). Next, a derivative of this virus, called FS97, carrying a capsid adapted to infect L929 cells [42] was used in coimmunoprecipitation experiments to assess the interaction of L* and RNase L in infected cells. L929 cells constitutively expressing the FLAG-muRNase L protein were generated by means of a lentiviral vector. These cells were infected for 16 h with FS97 (HA-L*) and total cells lysates were prepared and immunoprecipitated with anti-FLAG or anti-HA antibodies (Fig. 5). Western blot analysis of total cell lysates confirmed the expression of the expected proteins (Fig. 5B). When FLAG-muRNase L was immunoprecipitated from FS97-infected cells, a signal corresponding to the HA-L* protein was detected by immunoblot (Fig. 5A, lane 1). In the reciprocal experiment where HA-L* was pulled down, FLAG-muRNase L was detected in the immunoprecipitated complexes (Fig. 5A, lane 2). Control immunoprecipitations conducted either from FLAG-muRNase L-expressing cells infected with the parental KJ6 (L* WT) virus or from naive L929 cells infected with FS97 (HA-L*) failed to show co-immunoprecipitation of L* and RNase L (Fig. 5A, lanes 3–4 and 5–6). These results show that TMEV L* protein associates with murine RNase L in virus-infected cells.

### TMEV L* protein acts in a species-specific manner and interacts with the ankyrin domain of muRNase L

**Figure 6:**
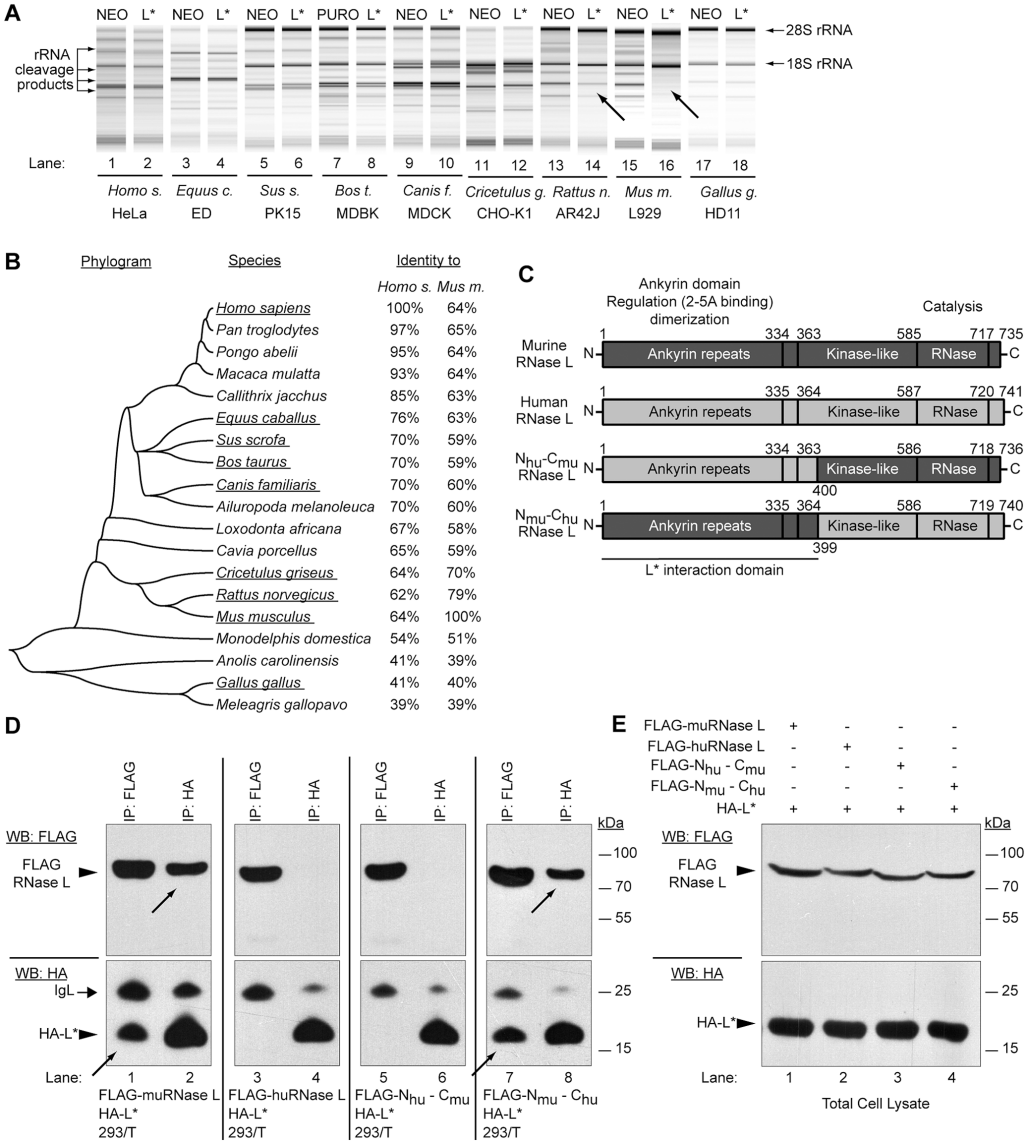
TMEV L* protein interacts with the N-terminal part of RNase L and is species specific. A. L* acts in a species-specific fashion: Indicated cell lines derived from different species were transduced with empty (Neo) or with L*-expressing lentiviruses and subsequently transfected with 2–5A to activate RNase L. RNA degradation was assessed by RNA chips. Arrows indicate samples where L* provided protection. B. Mid-point rooted neighbor-joining tree inferred from published RNase L protein sequences. Evolutionary distances were calculated using the Poisson correction method and a single bootstrapped consensus tree based on 1000 replicates is represented. Underlined species were tested for L*-mediated RNase L inhibition. C. Schematic representation of mouse, human and chimeric RNase L. Conserved domains of RNase L and their functions are indicated. D–E. 293/T cells were cotransfected with the chimeric constructs and total cell lysates were immunoprecipitated with anti-FLAG (RNase L) and anti-HA (L*) antibodies (D). Total cell lysates corresponding to 5% of the input used for immunoprecipitations were analyzed as controls (E). Samples were analyzed by SDS-PAGE and immunoblot analysis with indicated antibodies (WB:FLAG or WB:HA). Arrows point to coimmunoprecipitated proteins. IgL: immunoglobulin light chain.

To test whether L* inhibited RNase L of other species, L*-expressing cell lines were generated from human, equine, canine, rat, chicken, porcine, bovine, guinea pig and mouse cells, using a L*-expressing lentiviral construct. L* expression in the various cell lines was verified by immunocytochemistry (Fig. S6). Corresponding control cells were transduced with the empty vector. Then, endogenous RNase L activity was assessed by testing RNA degradation after 2–5A transfection. To our surprise, extensive RNA degradation was observed in L*-expressing cells as well as in control cells from all species with the exception of murine cells (Fig. 6A). RNase L inhibition was complete in mouse cells and intermediate in rat cells. Therefore, our data show that L* acts in a species-specific manner.

Next, we verified that L* interacted with murine RNase L but not with human RNase L. Therefore, 293/T cells were cotransfected with expression plasmids coding for HA-L* and for either murine or human FLAG-RNase L. As shown in Fig. 6D–E, L* co-immunoprecipitated with murine but not with human RNase L. These enzymes exhibit 64% aminoacid identity (Fig. 6B).

We took advantage of the lack of interaction between L* and the human RNase L to map the RNase L domain that interacts with L*. RNase L proteins are made of three conserved domains: the N-terminal “ankyrin” domain which regulates the endoribonuclease activity through 2–5A binding on ankyrin repeats and protein oligomerization, the central protein kinase-like, and the C-terminal domain which bears the catalytic site responsible for single-stranded RNA cleavage (Fig. 6C). Two plasmid vectors encoding chimeric FLAG-tagged RNase L proteins were constructed (Fig. 6C). The first encodes the N-terminal part (amino acids 1–400) of human RNase L followed by the C-terminal part of the murine RNase L (N_hu_-C_mu_) while the second encodes converse chimera (N_mu_-C_hu_). Coimmunoprecipitation between HA-L* and the FLAG-tagged chimeric RNase L were conducted in transfected 293/T cells. As shown, in Fig. 6D, L* interacted with the N_mu_-C_hu_ but not with the N_hu_-C_mu_ construct, suggesting that L* interacts with the N-terminal part (ankyrin domain) of murine RNase L.

### The interaction between Theiler's virus L* and murine RNase L is direct

**Figure 7:**
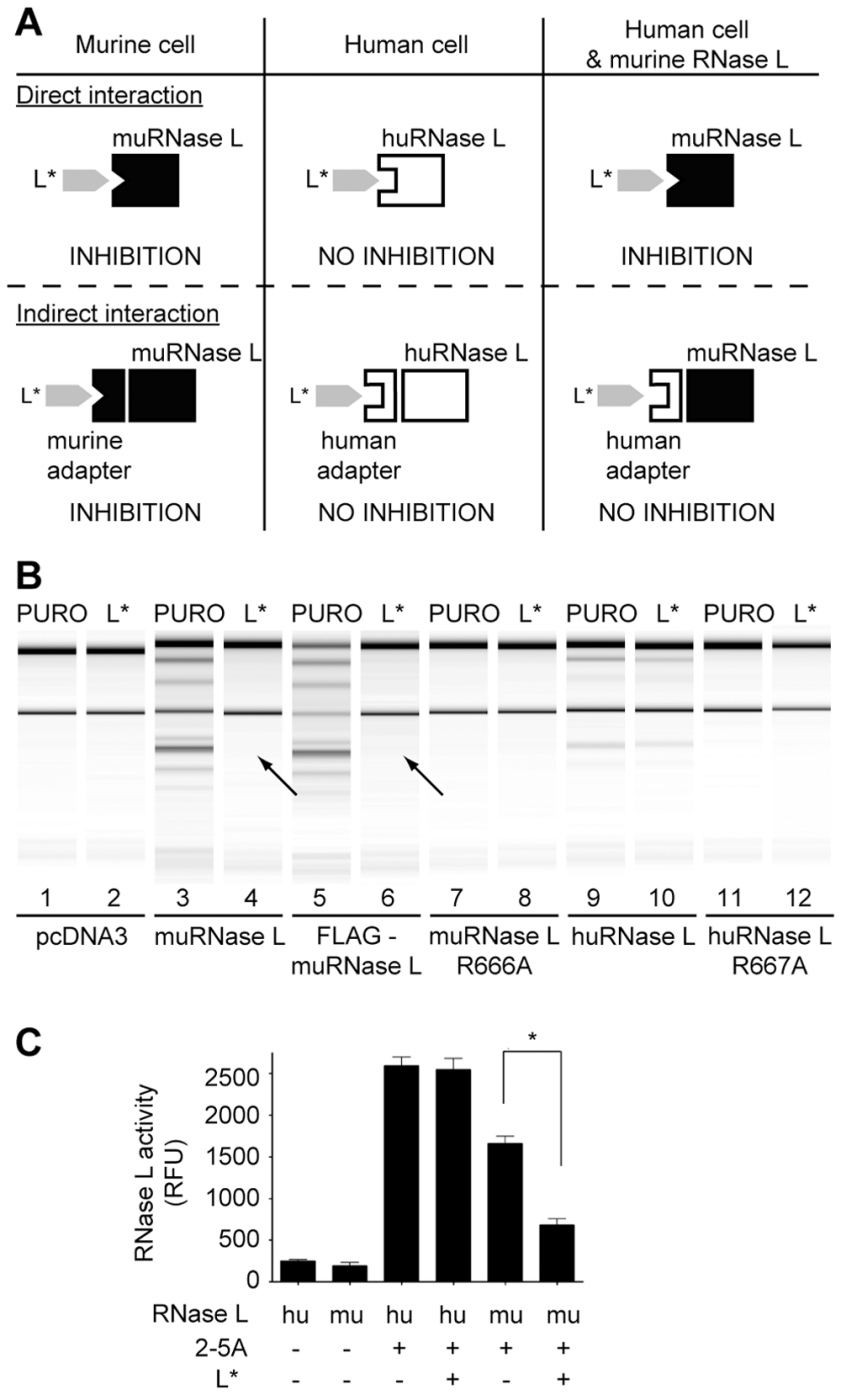
TMEV L* protein directly interacts with the murine RNase L. A. Cartoon showing expected inhibition of RNase L activity in the case of direct or indirect L*-RNase L interaction, in murine and human cells or in human cells expressing the murine RNase L. B. HeLa M cells stably expressing either an empty vector (Puro) or L* were transfected with pcDNA3 or with pcDNA3 derivatives expressing indicated RNase L constructs: wild-type (lanes 3–4), flagged (lanes 5–6) and catalytically inactive (R666A)(lanes 7–8) murine RNase L, or wild-type (lanes 9–10) and catalytically inactive (R667A)(lanes 11–12) human RNase L. Selection with 1 mg/ml of G418 was applied for 3 passages. Cells were then incubated with 100 U/ml human recombinant IFN-α-2a for 24 h. Poly(I:C) was then transfected 6 h before total cell RNA was extracted and analyzed on RNA chips. Arrows indicate lack of RNA degradation due to L* expression. C. L* inhibits RNase L *in vitro*. FRET assay performed with recombinant human (hu) RNase L at 100 nM or murine (mu) GST-RNase L at 180 nM, in the presence or absence of 2–5A at 2 nM and of recombinant His-tagged L* protein at 10 µg/ml. Histograms show mean relative fluorescence units (RFU) resulting from degradation of the FRET RNA probe.

Association of L* and murine RNase L in immunoprecipitates could either be direct or indirect, through an adapter protein intermediate. In the first case, the lack of inhibition of huRNase L by L* would result from the inability of L* to bind huRNase L. In the second case (adapter hypothesis), the lack of inhibition of huRNase L by L* would result from the inability of L* to bind the human adapter (Fig. 7A). Thus, when expressed in human cells, muRNaseL would be inhibited by L* in the case of direct but not in the case of indirect interaction, since these cells express the human adapter. As shown in Fig. 7B, L* expression clearly inhibited the activity of wild-type and of FLAG-tagged murine RNase L in human cells. Thus, we conclude that interaction between L* and muRNase L does not involve an intervening adapter protein.

### L* inhibits murine but not human RNase L *in vitro*

Murine and human RNase L were expressed and purified as recombinant proteins. Both enzymes displayed 2–5A-dependent activity *in vitro* (Fig. 7C), in a fluorescence energy transfer (FRET) assay based on the degradation of a labeled RNA probe [43]. Interestingly, murine but not human RNase L activity was significantly inhibited in this assay, after addition of purified His-tagged L* protein (Fig. 7C). This experiment confirms the lack of adaptor protein required for RNaseL inhibition by L* as well as the species-specificity of L* activity.

## Discussion

L* is an atypical picornavirus protein in that it is translated from an alternative ORF overlapping the ORF encoding the viral polyprotein. This “accessory” protein is important for the establishment of persistent CNS infections by TMEV [32], [34]. Given that a fraction of this protein is targeted to the mitochondrial outer membrane [39], [44], we aimed to confirm the anti-apoptotic role reported for L* in macrophages. Our data confirm the requirement of L* for virus growth in macrophages [35], [36], [45]. However, in contrast to previous reports [32], [38], our data revealed only marginal anti-apoptotic activity for L* in many different experimental settings. For instance, we failed to detect increased apoptosis in P388-D1, RAW264.7 or J774-1 macrophages infected with the L* mutant viruses. Also, ectopic expression of L* in J774-1 or RAW264.7 cells did not decrease apoptosis after treatment of the cells with apoptosis-inducing stimuli such as ultraviolet light, etoposide, hydrogen peroxyde, staurosporine or viral infection. In contrast, our data show a prominent role of L* in suppression of RNase L activity. Both in J774-1 cells and in primary macrophages from wild-type mice, replication of L*-mutants was significantly reduced as compared to that of the wild-type virus. In contrast, in RNase L^−/−^ primary macrophages, L*-mutant viruses replicated to wild-type levels. Moreover, in RNase L^+/+^ macrophages, ectopic expression of L* restored replication of mutant viruses to wild-type levels. Taken together, these data suggest that RNase L activity is a major cause of L*-mutant virus replication inhibition in macrophages and that ectopic expression of L* allows to rescue the replication of mutant viruses, through RNase L antagonism. Interestingly, antagonism of RNase L by L* was not restricted to macrophages but occurred in other cell types after priming of these cells with IFN. Accordingly, macrophages turned out to have elevated basal OAS expression levels as compared to fibroblasts but, after IFN treatment, OAS expression was upregulated in fibroblasts and replication of the L*-mutant viruses was also restricted in these cells.

Unfortunately, the influence of RNase L inhibition by L* could not be assessed in experimental mouse infection. Indeed, available RNase L^−/−^ mice are on the H-2^b^ haplotype [46] and are therefore highly resistant to TMEV infection since they can mount a prominent cytolytic T lymphocyte response against an immunodominant TMEV capsid epitope [47]–[49]. However, our data clearly document the importance of RNase L antagonism by L* in primary macrophages. Macrophages are known to be relevant to TMEV infection as these cells were shown to bear the major viral load during persistence of the virus in the CNS [28].

L* inhibits the OAS/RNase L pathway by interacting directly with the effector enzyme of the pathway (i.e. RNase L). This was confirmed in an *in vitro* assay in the absence of any other viral or cellular protein. L* appears to interact with the ankyrin domain of RNase L. Structural data suggest that 2–5A binding to ankyrin repeats triggers the dimerization, and further the oligomerization, process that leads to RNase L activation [6]. By binding the ankyrin domain, L* could either inhibit 2–5A binding to RNase L or prevent the dimerization that was shown to occur during activation of the endoribonuclease [4], [6], [50]. Further experiments, including precise mapping of the interaction site are needed to gain insights in the molecular mechanisms engaged by L* to inhibit RNase L activation.

Several viral proteins were shown to interfere with the OAS/RNase L pathway. Proteins like influenza A NS1 are believed to act by sequestering dsRNA and therefore by preventing OAS enzyme activation [51]. By acting upstream of the pathway, such proteins have a broader spectrum of action. Indeed, hiding dsRNA is also expected to prevent other events triggered by dsRNA, such as the activation of PKR or of the RIG-like helicases. L* acts at the opposite end of the pathway. The advantage of targeting the effector enzyme is that leakiness of the system is less likely. Accordingly, the antagonism of RNase L by L* looked very potent. Moderate levels of L* expression from retroviral vectors completely inhibited RNA degradation in infected cells or in cells transfected with 2–5A. The fact that Theiler's virus devotes a protein to antagonize the RNase L effector suggests that this specific pathway is particularly important in the control of Theiler's virus infection.

Interestingly, L* inhibition of RNase L activity turned out to be strictly species-specific. L* inhibited the activity of murine but not of human, equine, canine porcine, bovine, guinea pig or chicken RNase L. Saffold virus is a recently discovered human virus closely related to TMEV [52]–[54]. The numerous isolates of this virus sequenced to date lack the L* coding region and thus likely fail to inhibit RNase L activity by this mechanism. This suggests that RNase L is either a more critical IFN effector in mice than in humans, or that RNase L activity is particularly prominent in specific cell types that are infected by TMEV more than by Saffold virus. In this respect, it is interesting to note that mouse hepatitis virus (MHV) was recently shown to interfere with RNase L activation in macrophages by triggering the cleavage of 2–5A with a virally-encoded 2′,5′-phosphodiesterase [15]. TMEV and MHV share a marked tropism for macrophages. It is thus tempting to speculate that the antiviral activity of RNase L is particularly prominent in macrophages which fits with the observation that these cells contain higher basal levels of OAS enzymes and likely mount a faster and more potent RNase L response than other cells.

In summary, we report a new mechanism of RNase L inhibition by a viral protein occurring through direct protein-protein interaction. It is interesting to note that a single region of Theiler's virus RNA encodes two proteins, L and L* that both counteract the IFN pathway to allow persistence of the virus: the former through IFN gene transcription inhibition and the latter through inhibition of an IFN-induced effector.

## Materials and Methods

### Ethics statement

Handling of mice (agreement LA1230472) and experimental procedures were conducted in accordance with the EEC directive 86/609/CEE and the related Belgian law of April 6th 2010. The study and protocol used in this study were approved by the ethics committee of the University of Louvain under the agreement # 2010/UCL/MD/031.

### Cells

BHK-21 cells were cultured in Glasgow-minimal essential medium supplemented with 10% newborn calf serum and 2.95 g/l of tryptose phosphate broth. HeLa M cells were cultured in Dulbecco modified Eagle medium (DMEM) supplemented with 10% fetal calf serum (FCS). J774-1 cells were cultured in DMEM supplemented with 10% FCS and 10 mM HEPES, pH 7.5. Peritoneal macrophages from 129/Sv, RNase L^−/−^ and C57BL/6 mice [46] were harvested 4 days after i.p. inoculation of 1.5 ml of sterile 10% Thioglycolate Medium, Brewer Modified (Becton Dickinson BBL, Cat N°: 211716). Cells were washed by centrifugation at 300× g for 5 min and resuspended in RPMI containing 10% FCS, 25 mM HEPES, pH 7.5 and 2 mM L-glutamine. Macrophages were then plated for 3 and 4 days prior to infection. Purity of macrophages (>95%) was measured by Mac-1 staining. All media were supplemented with 100 U/ml of penicillin and 100 µg/ml of streptomycin.

### Viruses and vectors

TMEV derivatives were produced by electroporation of BHK-21 cells as previously described [55], [56] with the genomic RNA transcribed *in vitro* from plasmids carrying the corresponding full-length viral cDNA (Table 1). Viruses were derived from the DA1 molecular clone (Genbank accession JX443418). A recombinant TMEV cDNA, named pFS96, was constructed, carrying the extra sequence coding for the HA epitope at the N-terminus of L*: 5′ GGT ACC CGT ACG ACG TTC CGG ACT ACG CGC TGC TTG TAA GCA CGG 3′, between nucleotides 1081 and 1082 of pTMDA1. The corresponding virus, FS96, produced by reverse genetics gave titers and plaque sizes similar to those of the parental DA1 virus (DA1: 7.6 10^7^ PFU/ml; FS96: 6.4 10^7^ PFU/ml). DA1 mutants carrying stop codon mutations in L* were described previously. In these viruses, mutations L* 1–12 (virus OV84) and L* 1–92 (virus OV42) prevented the expression of full-length L* protein but failed to affect the translation of the main viral open reading frame encoding the polyprotein. Both mutations affected the ability of the virus to establish persistent infections of the mouse CNS [34]. For L929 cell infection, DA1 derivatives carrying capsid mutation that enhance L929 cell infection were used [42]. These viruses, KJ6 (L* WT), FS57 (L* 1–92), and FS97 (HA-L*) were generally used at a MOI of 2 PFU per cell as this MOI usually yields almost 100% of infection [42]. In L929 cells infected with these viruses, cytopathic effect becomes visible from 6 hours post-infection. However, viral replication was found to peak in these cells between 12 and 20 h post-infection. For macrophage infection, derivatives carrying a macrophage-adapted capsid (mutations VP2 G162S, VP1 V256E and VP1 S257Y) were used: VV18 (L* WT), TM770 (L* 1–92) and FS58 (L* 1–12). These viruses were further purified as follows: SDS was added to viral stocks (BHK-21 cell supernatants) to a final concentration of 1% and incubated for 1 h at room temperature. Insoluble materials were removed by centrifugation for 15 min at 1000× g and viruses were pelleted by centrifugation at 80,000× g for 14 h at 20°C. Viruses were resuspended in 300 µl of Tris-HCl 10 mM, pH 7.5 and centrifuged at 96,000× g for 18 h at 4°C, through a 30% sucrose cushion. Viruses were resuspended in 300 µl Tris-HCl, 10 mM pH 7.5, dialyzed against the same buffer using Slide-A-Lyser Dialysis Cassette 10,000 MWCO (Pierce) and titrated on BHK-21 cells by plaque assay. For macrophage infection, viruses were typically used at a MOI of 20 PFU per cell to reach near 100% infection and viral replication was assessed between 6 and 12 hours post-infection, before extensive cell degradation.

**Table 1:** Plasmids carrying TMEV full length cDNA used in this study.

Mutation/tag	WT capsid	L929 cell-adapted^1^	macrophage-adapted^2^
None (L* WT)	pTMDA1	pKJ6	pVV18
HA-L* WT	pFS96	pFS97	
L* (1–92)		pFS57	pTM770
L* (1–12)			pFS58

1 Viruses derived from these plasmids carry point mutations in the capsid that increase the efficiency of L929 cell infection.

2 Viruses derived from these plasmids carry point mutations in the capsid that increase the efficiency of macrophage infection.

Retroviral vectors were derived from pQCXIN and pQCXIP (Clontech). Lentiviral vectors were derived from pCCLsin.PPT.hPGK.GFP.pre. [57]. Viral particles were pseudotyped with the glycoprotein of vesicular stomatitis virus (VSV-G). Transduced cells were selected with G418 (1 mg/ml) or puromycin (2 µg/ml). Transgene expression was verified by indirect immunufluorescence or immunoblot analysis. Expression vectors used in this study are summarized in Table 2.

**Table 2:** Expression plasmids used in this study.

Name	Expressed gene	Resistance^1^	Type (parental vector)
pQCXIN	-	Neo	Retroviral (MuLV)
pFS24	L*	Neo	Retroviral (pQCXIN)
pFS117	HA-L*	Neo	Retroviral (pQCXIN)
pFS118	HA-L*-FS	Neo	Retroviral (pQCXIN)
pQCXIP	-	Puro	Retroviral (MuLV)
pFS27	L*	Puro	Retroviral (pQCXIP)
pTM898	-	Neo	Lentiviral (HIV)
pFS119	L*	Neo	Lentiviral (pTM898)
pTM900	-	Hygro	Lentiviral (HIV)
pFS174	FLAG-muRNase L	Hygro	Lentiviral (pTM900)
pcDNA3	-	Neo	Plasmid
pTM667-8	L*	Neo	Plasmid (pcDNA3)
pFS111	HA-L*	Neo	Plasmid (pcDNA3)
pFS164	muRNase L	Neo	Plasmid (pcDNA3)
pFS165	FLAG-muRNase L	Neo	Plasmid (pcDNA3)
pFS172	muRNase L R666A	Neo	Plasmid (pcDNA3)
p-huRNase L	huRNase L	Neo	Plasmid (pcDNA3)
pFS183	FLAG-huRNase L	Neo	Plasmid (pcDNA3)
p-huRNase L R667A	huRNase L R667A	Neo	Plasmid (pcDNA3)
pFS187	FLAG-Nhu-Cmu RNase L	Neo	Plasmid (pcDNA3)
pFS188	FLAG-Nmu-Chu RNase L	Neo	Plasmid (pcDNA3)
pFS105	6×His-L*	Ap	Bacterial (pET15b)
pFS178	GST-muRNaseL	Ap	Bacterial (pDEST15)

1 Neo: G418/Geneticin, puro: puromycin, hygro: hygromycin.

### RNA isolation, RNA chips and real-time PCR

RNA was isolated by the method of Chomczinsky and Sacchi [58]. Reverse transcription reactions and real-time PCR were performed as previously described [59]. Primers used for amplification are presented in Table 3. RNA degradation was assessed by running RNA samples either on conventional 1% agarose gels in Tris-sodium acetate-EDTA (TAE) buffer or on RNA nano 6000 microfluidics chips run on a 2100 Bioanalyzer (Agilent Technologies).

**Table 3:** Primers used for real-time PCR.

Name	Sequence (5′→3′)	Use
TM346 Fwd	GCC GCT CTT CAC ACC CAT	qPCR TMEV
TM347 Rev	AGC AGG GCA GAA AGC ATC AC	qPCR TMEV
TM638 Fwd	GGA TGC CTG GGA GAG AAT CG	qPCR Oasl2
TM639 Rev	TCG CCT GCT CTT CGA AAC TG	qPCR Oasl2
TM747 Fwd	TCC GAT GGA TGG GAG AGT CA	qPCR muRNase L
TM748 Rev	AAA GGA TGG CCA AGC AGG TC	qPCR muRNase L
TM646 Fwd	TTG TGT GGA CCT GGA CGA TG	qPCR L*
TM647 Rev	CCG CTG GCA GAC AAA TCA AT	qPCR L*
TM421 Fwd	AGA GGG AAA TCG TGC GTG AC	qPCR β-actin
TM422 Rev	CAA TAG TGA TGA CCT GGC CGT	qPCR β-actin

### Immunofluorescence

Cells grown on poly-L-lysine-treated coverslips were fixed for 15 min with paraformaldehyde 4% in PBS. Cells were then permeabilized for 5 min with Triton X-100 0.1% in PBS and unspecific antigens were blocked for 1 h using 2% normal goat serum (Sigma) in Protein Block Serum Free solution (DAKO, X0909). Cells were then incubated for 1 hour with primary antibodies diluted in the same buffer at a dilution of 1∶800 (anti-L*, rabbit polyclonal) or 1∶10 (anti VP1-F12B3, mouse monoclonal). After extensive washes with Tween 20 0.1% in PBS, species-matched AlexaFluor-conjugated secondary antibodies (Molecular Probes, A11020, A11008) were added at a dilution of 1∶800 in Antibody Diluent with Background Reducing Components (DAKO, S3022) for one additional hour. Coverslips were mounted on slides with Mowiol supplemented with DABCO.

### Measurement of apoptosis

Caspase 3/7 activity was measured using Caspase-Glo 3/7 Assay (Promega). Briefly, 1.5 10^4^ J774-1 cells grown in 96 well plates were infected in a volume of 50 µl at 20PFU per cell. At indicated times, plates were frozen and thawed. The luminogenic substrate was added for 1 h and light emission was measured. For *in situ* TUNEL assay, cells grown on coverslips were infected and labeled using the In Situ Cell Death Detection Kit, fluorescein (Roche) following manufacturer's instructions.

### Mice

To test FS96 persistence, female 3-week-old FVB/N (Charles River Laboratories) were anesthetized and infected intracranially by injection of 40 µl of serum-free medium containing 10^5^ PFU of the virus. Control mice were injected with 40 µl of serum-free culture medium. At 45 days post-infection, total RNA was extracted from the spinal cords of infected mice as previously described [59].

### Coimmunoprecipitation assays

Infected or transfected cells were washed with cold phosphate-buffered saline (PBS) and harvested in lysis buffer (Tris-HCl 50 mM pH 8, NaCl 100 mM, NP40 0.5%, EDTA 2 mM, PMSF 1 mM, protease (Sigma) and phosphatase (Calbiochem) inhibitors). Cells were disrupted by 10 passages through a 21G needle and lysates were centrifuged at 12,000× g for 10 min at 4°C. Supernatants were pre-cleared using 50 µl bed-volume of Protein A/G UltraLink Resin (Pierce) for one hour at 4°C and centrifuged supernatants were reacted with anti-FLAG M2 (Sigma) or anti-HA (Covance) antibodies with gentle agitation for 2 hours at 4°C. Protein A/G UltraLink Resin was added and the lysates were incubated for an additional 2 h at 4°C. The beads were then washed 4 times with lysis buffer without inhibitors (with the exception of PMSF). Immunoprecipitated proteins were detected using SDS-PAGE and immunoblot analysis. As a control of protein expression in cells used for coimmunoprecipitation experiments, total cell lysate corresponding to 5% of the input used for immunoprecipitation was also analyzed by immunoblot.

### Poly(I:C) and 2–5A transfections

Cells plated in 24 well-plates were transfected with 2.5 µg poly(I:C) per ml (Amersham-Pharmacia) or 5 µM unfractionated 2–5A (prepared as described in [19], [60]) using 2 µl Lipofectamine 2000 transfection reagent according to the manufacturer's protocol (Invitrogen).

### L* and RNase L recombinant protein expression and purification

The plasmid L*-pET15b was expressed in shuffle T7 express *E. coli* (NEB, Inc.). Bacteria were grown in LB containing 100 µg/ml ampicillin at 37°C, and shaked at 250 rpm until the OD600 nm reached 0.7. The cultures were cooled on ice, induced using 0.5 mM IPTG and further grown for 6 h at 20°C. Cells were harvested and washed with ice cold 20 mM HEPES pH 8.2 containing 150 mM NaCl and lysed by sonication (20×10 s) in buffer A (20 mM HEPES pH 8.2, 10% glycerol, 14.2 mM beta mercaptoethanol, and 200 mM NaCl) containing 10 units/ml benzonase, protease inhibitor cocktail (Roche) and 100 µM PMSF. Lysates were clarified by centrifugation at 10,000× g for 30 min at 4°C, diluted 5-fold and bound to SP Sepharose FF cation exchange resin (GE Lifesciences) packed in XK 15 mm×10 mm column. The column was washed with binding buffer A until the basal OD280 nm was constant. The bound proteins were eluted with a 0.2–2 M gradient of NaCl in buffer A. The fractions were analyzed by SDS page (L* eluted at >500 mM NaCl). The peak fractions containing L* were pooled and diluted 1∶3 in buffer A and bound with Ni-NTA resin, washed with buffer A containing 10 mM imidazole and eluted with 150 mM imidazole in buffer A. Fractions were loaded on 12% SDS-PAGE and stained with Gel-Code Blue (Promega). The yield was ∼0.25 mg/L.

Human RNase L was produced as described in Dong et al., [61]. Murine RNase L was cloned as a N-terminal GST fusion in the pDEST15 vector, using a LR gateway reaction (Invitrogen). The recombinant protein was produced in *E. coli*, as described [4].

### FRET assays for RNase L activity

RNase L activity was determined using fluorescence resonance energy transfer (FRET) assays as described previously [43]. Briefly, the recombinant murine (180 nM) or human RNase L (100 nM) were pre-incubated with purified L* (10 µg/ml) for 10 min on ice followed by the addition of trimeric 2–5A (2 nM) and FRET probe (a 36 nucleotide synthetic oligoribonucleotide [5′ (6-FAM-UUAUCAAAUUCUUAUUUGCCCCAUUUUUUUGGUUUA-BHQ-1)-3′] corresponding to a sequence of respiratory syncytial virus [43]. The reactions (50 µl) contained 100 nM FRET probe, 25 mM Tris–HCl (pH 7.4), 100 mM KCl, 10 mM MgCl_2_, 100 µM ATP, and 7.2 mM 2-mercaptoethanol. The plates were incubated at 20°C protected from light. Fluorescence was measured with a Wallac 1420 fluorimeter (Perkin–Elmer LAS Inc., USA) (excitation 485 nm/emission 535 nm with a 0.1 s integration time).

### Measuring 2–5A levels in cells

The L929 cells stably expressing L* (L929-L*) or transduced with the empty vector (L929-NEO) were transfected with poly(I :C) for different times. Cells were then harvested and lysed by addition of NP-40 buffer [50 mM Tris–HCl (pH 7.2), 150 mM NaCl, 1% NP-40, 200 µM NaVO_3_, 2 mM EDTA, 5 mM MgCl_2_ and 5 mM DTT] that was pre-heated at 95°C for 3 min. The cell suspension was heated to 95°C for 7 min. Exogenous poly(I:C) was added in the lysis buffer as a control for activation of OAS enzyme in L929-L* or L929-NEO cells after cell lysis. Cell debris was removed by centrifugation and the supernatants were applied to Vivaspin 500 centrifugal filter devices (3 kDa molecular weight limit, Vivascience) and centrifuged at 10,000× g for 45 min at 4°C. The samples were stored at −80°C until use. Level of 2–5A was determined by FRET assays [43].

### Statistical analysis

Results are expressed as means +/− standard deviations (SD). Statistical analyses were performed on triplicate experiments using the one-tailed Mann-Whitney test. NS is indicative of p-values higher than 0.05; * is indicative of p-values of 0.05.

## Supporting Information

Figure S1 Virus yield and apoptosis in L*-mutant and wild-type virus infected cells. J774-1 macrophages grown on coverslips were infected for 10 h with 20 PFU per cell of VV18 (L*-WT), TM770 (L* 1–92) or FS58 (L* 1–12) viruses or were mock-infected. Detection of nicked DNA in apoptotic cells was performed using an *in situ* TUNEL assay. A. Detection of apoptotic cells by *in situ* TUNEL assay (green) and intracellular immunodetection of viral capsid protein VP1 (red). Macrophage nuclei were stained with Hoechst 33342 (blue). B. Histogram showing the percentage of TUNEL-positive cells, counted under an epifluorescence microscope (n>700).
(TIF)

Figure S2 Absence of RNase L rescues the replication of L*-mutant viruses in primary macrophages. Peritoneal macrophages isolated from RNase L^−/−^ and RNase L^+/+^ mice were grown on coverslips for 4 days and infected with purified viruses for 9 hours at a multiplicity of infection of 20 PFU per cell. Replication of wild-type (L* WT) or L*-mutant (L* 1–92 and L* 1–12) was revealed by intracellular detection of viral capsid protein VP1 (red). Macrophage nuclei were stained with Hoechst 33342 (blue).
(TIF)

Figure S3 ***Oasl2*** **and** ***RNase L*** **gene expression analysis in different murine cells, as determined by quantitative RT-PCR.** Cells were treated with 5 U/ml of IFN-β when specified and total cellular RNA was harvested, reverse transcribed using random hexamers. Histograms show the cDNA copy number of *Oasl2* (A) and of *RNase L* (B) transcripts measured by quantitative RT-PCR and normalized for each sample to β-actin cDNA. Standard curves for *Oasl2*, *RNase L* and *β-actin* amplification were obtained by serial dilutions of plasmids carrying the corresponding target amplicons. Values are means +/− SD for an experiment performed in triplicate.
(TIF)

Figure S4 Ectopic expression of L* protein rescues the replication of L*-mutants but does not inhibit 2–5A production. A. Expression of L* rescues the replication of a TMEV L*-mutant: IFN-primed clones of L929 cells stably expressing L* (L929-L*) or transduced with the empty vector (L929-NEO) were infected with KJ6 (L* WT) or FS57 (L* 1–92). Histograms show viral genome quantification by quantitative RT-PCR in three independent cell clones infected for 16 h with 2 PFU per cell of the indicated viruses. VP1 capsid immunostaining (data not shown) confirmed the higher replication of wild-type virus in cells carrying the empty vector and equal replication of L*-mutant and wild-type viruses in cells expressing L*. B. J774-1 macrophages were transduced with an empty lentiviral vector (J774-NEO) or with the same vector expressing L* (J774-L*). These cells were then either mock-infected (white columns) or infected with 10 PFU per cell of TM770 (L*1–92). RNA collected 10 hours post-infection was analyzed on a native 1% agarose gel to assess RNA degradation (left panel) and used for RT-qPCR analysis of viral replication (right panel). Values for mock samples were lower than the detection limit (10 cDNA copies). C. Expression of L* does not affect 2–5A production. L929-L* and L929-NEO cells were transfected with 2 µg/ml poly(I:C) for the indicated times and cells were harvested as described (see Materials and Methods). The 2–5A concentration after extraction from the cells was measured by FRET assays by using a standard curve (*R^2^* = 0.9963) with known concentrations of authentic 2–5A. 2–5A production was not significantly different in L*-expressing (L929-L*) and control (L929-NEO) cells. As a control, poly(I:C) was added to the lysis buffer and used on untreated cells to monitor possible OAS activation after cell lysis [indicated as “poly(I:C) in buffer”].
(TIF)

Figure S5 Recombinant Theiler's virus expressing a HA-tagged L* protein persists in the CNS of susceptible mice. Histograms show the results of viral genome quantification by RT-PCR performed on RNA extracted from spinal cords of FVB/N mice 45 days after intracranial infection with 10^5^ PFU of virus (n = 4). The background level of virus genome detection in mock samples (4 logs lower) likely stems from contamination of the samples at the time of tissue dissection. FS96 virus identity was verified by direct sequencing of RT-PCR products obtained from cDNA derived from spinal cords.
(TIF)

Figure S6 Expression of L* in cell lines from different species. Immunofluorescent labeling of L* (green) and nuclear staining (blue) of cells transduced with a lentiviral vector (pFS119) co-expressing L* and the geneticin/G418 resistance gene. Immunolabelings show that, after G418 selection, almost all the cells express detectable amounts of L*.
(TIF)
