# Supplementary figures and images for: Evasion of Antiviral Innate Immunity by Theiler's Virus L* Protein through Direct Inhibition of RNase L
Source: PLoS Pathog. 2013 Jun 27;9(6):e1003474. doi: 10.1371/journal.ppat.1003474 (PMC3694852; doi:10.1371/journal.ppat.1003474)

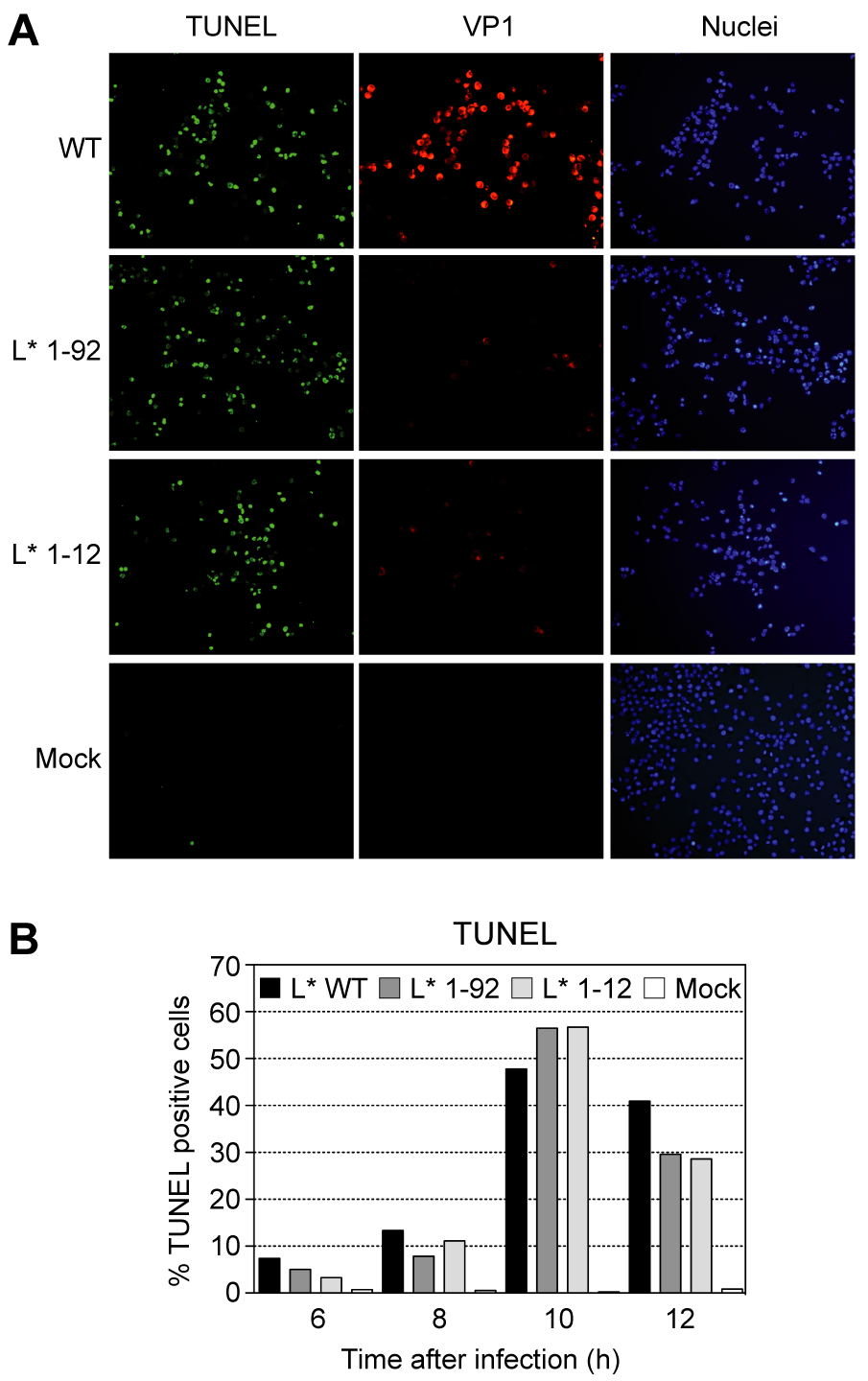

Supplement: Figure S1 — Virus yield and apoptosis in L*-mutant and wild-type virus infected cells. J774-1 macrophages grown on coverslips were infected for 10 h with 20 PFU per cell of VV18 (L*-WT), TM770 (L* 1–92) or FS58 (L* 1–12) viruses or were mock-infected. Detection of nicked DNA in apoptotic cells was performed using an in situ TUNEL assay. A. Detection of apoptotic cells by in situ TUNEL assay (green) and intracellular immunodetection of viral capsid protein VP1 (red). Macrophage nuclei were stained with Hoechst 33342 (blue). B. Histogram showing the percentage of TUNEL-positive cells, counted under an epifluorescence microscope (n>700). (TIF) [file ppat.1003474.s001.tif]

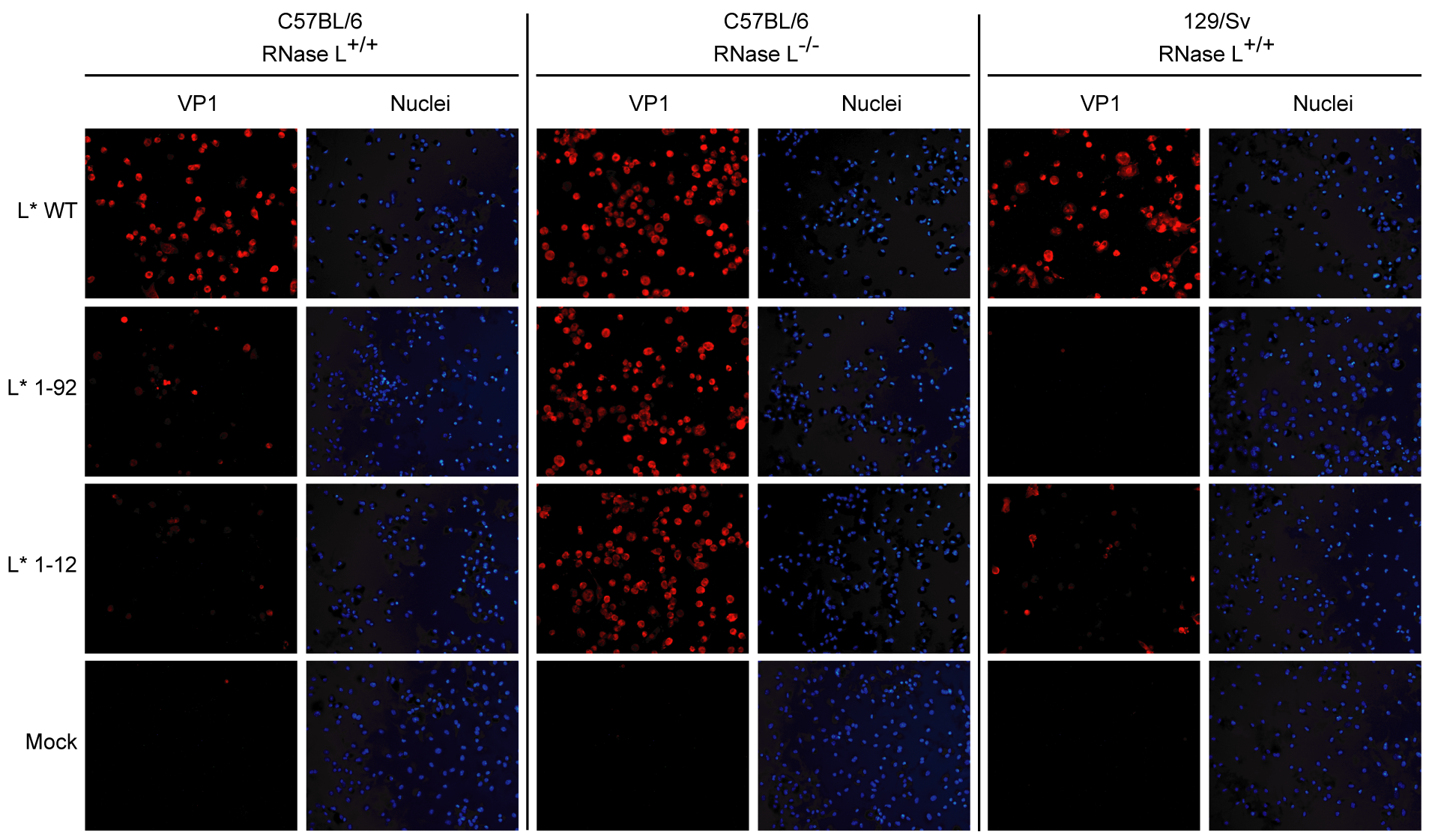

Supplement: Figure S2 — Absence of RNase L rescues the replication of L*-mutant viruses in primary macrophages. Peritoneal macrophages isolated from RNase L−/− and RNase L+/+ mice were grown on coverslips for 4 days and infected with purified viruses for 9 hours at a multiplicity of infection of 20 PFU per cell. Replication of wild-type (L* WT) or L*-mutant (L* 1–92 and L* 1–12) was revealed by intracellular detection of viral capsid protein VP1 (red). Macrophage nuclei were stained with Hoechst 33342 (blue). (TIF) [file ppat.1003474.s002.tif]

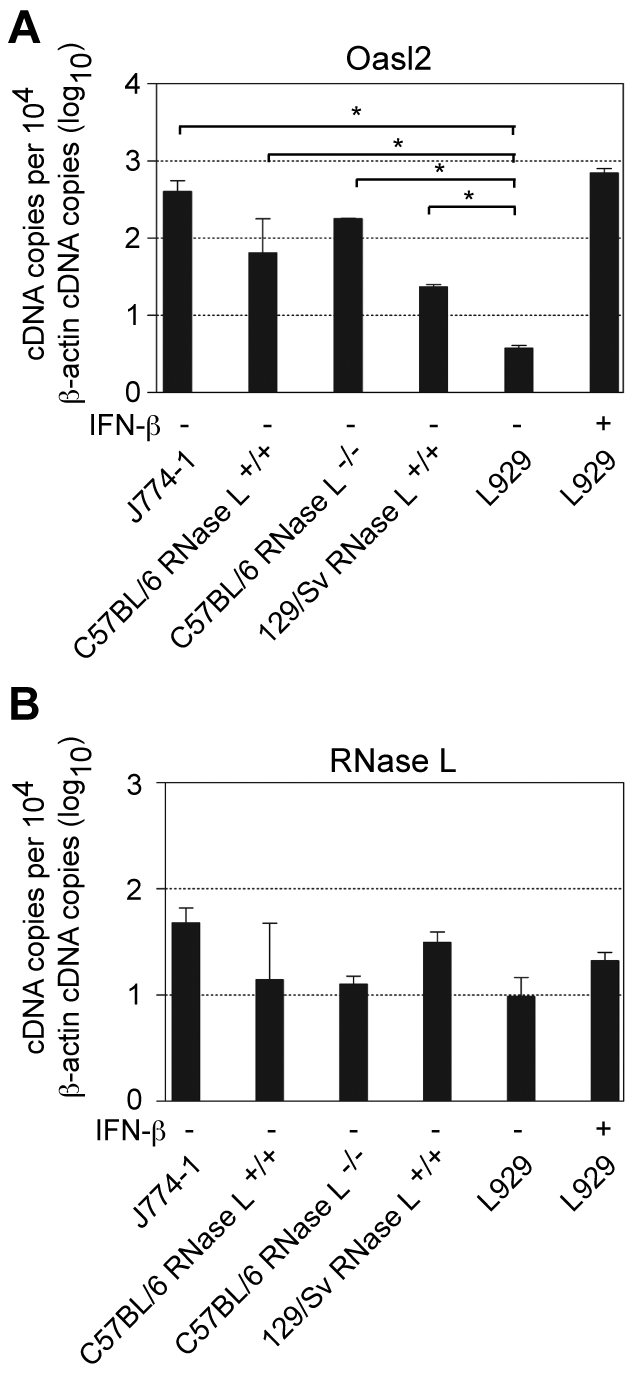

Supplement: Figure S3 — Oasl2 and RNase L gene expression analysis in different murine cells, as determined by quantitative RT-PCR. Cells were treated with 5 U/ml of IFN-β when specified and total cellular RNA was harvested, reverse transcribed using random hexamers. Histograms show the cDNA copy number of Oasl2 (A) and of RNase L (B) transcripts measured by quantitative RT-PCR and normalized for each sample to β-actin cDNA. Standard curves for Oasl2, RNase L and β-actin amplification were obtained by serial dilutions of plasmids carrying the corresponding target amplicons. Values are means +/− SD for an experiment performed in triplicate. (TIF) [file ppat.1003474.s003.tif]

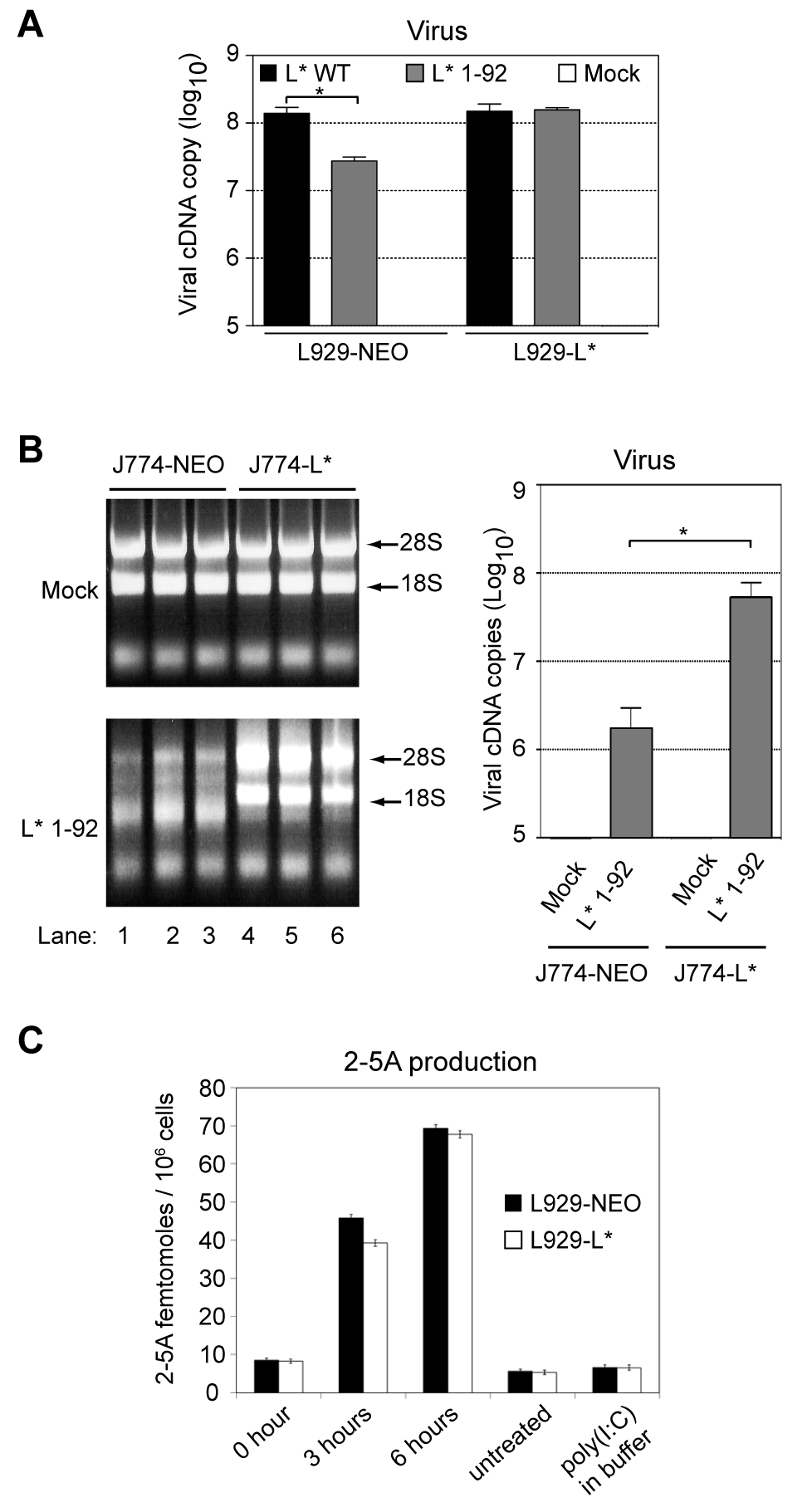

Supplement: Figure S4 — Ectopic expression of L* protein rescues the replication of L*-mutants but does not inhibit 2–5A production. A. Expression of L* rescues the replication of a TMEV L*-mutant: IFN-primed clones of L929 cells stably expressing L* (L929-L*) or transduced with the empty vector (L929-NEO) were infected with KJ6 (L* WT) or FS57 (L* 1–92). Histograms show viral genome quantification by quantitative RT-PCR in three independent cell clones infected for 16 h with 2 PFU per cell of the indicated viruses. VP1 capsid immunostaining (data not shown) confirmed the higher replication of wild-type virus in cells carrying the empty vector and equal replication of L*-mutant and wild-type viruses in cells expressing L*. B. J774-1 macrophages were transduced with an empty lentiviral vector (J774-NEO) or with the same vector expressing L* (J774-L*). These cells were then either mock-infected (white columns) or infected with 10 PFU per cell of TM770 (L*1–92). RNA collected 10 hours post-infection was analyzed on a native 1% agarose gel to assess RNA degradation (left panel) and used for RT-qPCR analysis of viral replication (right panel). Values for mock samples were lower than the detection limit (10 cDNA copies). C. Expression of L* does not affect 2–5A production. L929-L* and L929-NEO cells were transfected with 2 µg/ml poly(I:C) for the indicated times and cells were harvested as described (see Materials and Methods). The 2–5A concentration after extraction from the cells was measured by FRET assays by using a standard curve (R2 = 0.9963) with known concentrations of authentic 2–5A. 2–5A production was not significantly different in L*-expressing (L929-L*) and control (L929-NEO) cells. As a control, poly(I:C) was added to the lysis buffer and used on untreated cells to monitor possible OAS activation after cell lysis [indicated as “poly(I:C) in buffer”]. (TIF) [file ppat.1003474.s004.tif]

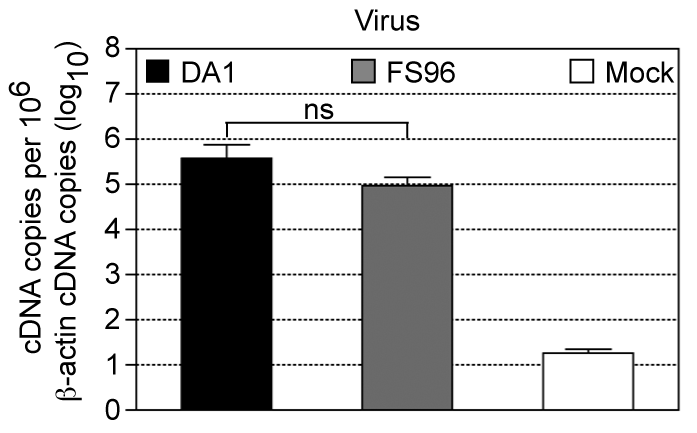

Supplement: Figure S5 — Recombinant Theiler's virus expressing a HA-tagged L* protein persists in the CNS of susceptible mice. Histograms show the results of viral genome quantification by RT-PCR performed on RNA extracted from spinal cords of FVB/N mice 45 days after intracranial infection with 105 PFU of virus (n = 4). The background level of virus genome detection in mock samples (4 logs lower) likely stems from contamination of the samples at the time of tissue dissection. FS96 virus identity was verified by direct sequencing of RT-PCR products obtained from cDNA derived from spinal cords. (TIF) [file ppat.1003474.s005.tif]

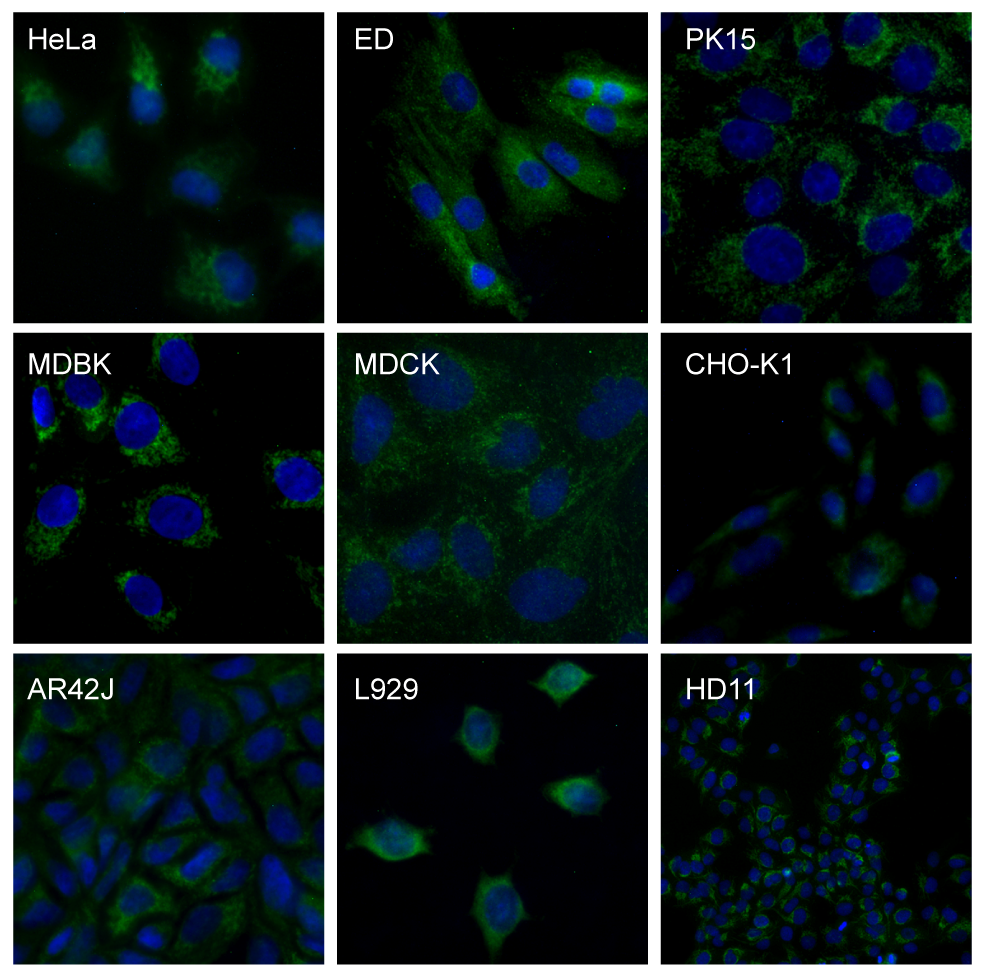

Supplement: Figure S6 — Expression of L* in cell lines from different species. Immunofluorescent labeling of L* (green) and nuclear staining (blue) of cells transduced with a lentiviral vector (pFS119) co-expressing L* and the geneticin/G418 resistance gene. Immunolabelings show that, after G418 selection, almost all the cells express detectable amounts of L*. (TIF) [file ppat.1003474.s006.tif]
